# The developmentally dynamic microRNA transcriptome of *Glossina pallidipes* tsetse flies, vectors of animal trypanosomiasis

**DOI:** 10.1093/bioadv/vbab047

**Published:** 2021-12-28

**Authors:** Careen Naitore, Jandouwe Villinger, Caleb K Kibet, Shewit Kalayou, Joel L Bargul, Alan Christoffels, Daniel K Masiga

**Affiliations:** 1 International Centre of Insect Physiology and Ecology (icipe), P.O. Box 30772, Nairobi 00100, Kenya; 2 Department of Biochemistry, Jomo Kenyatta University of Agriculture and Technology (JKUAT), P.O. Box 62000, Nairobi 00200, Kenya; 3 South African Medical Research Council Bioinformatics Unit, South African National Bioinformatics Institute (SANBI), University of the Western Cape, Bellville 7530, South Africa

## Abstract

**Summary:**

MicroRNAs (miRNAs) are single stranded gene regulators of 18–25 bp in length. They play a crucial role in regulating several biological processes in insects. However, the functions of miRNA in *Glossina pallidipes*, one of the biological vectors of African animal trypanosomosis in sub-Saharan Africa, remain poorly characterized. We used a combination of both molecular biology and bioinformatics techniques to identify miRNA genes at different developmental stages (larvae, pupae, teneral and reproductive unmated adults, gravid females) and sexes of *G. pallidipes*. We identified 157 mature miRNA genes, including 12 novel miRNAs unique to *G. pallidipes*. Moreover, we identified 93 miRNA genes that were differentially expressed by sex and/or in specific developmental stages. By combining both miRanda and RNAhybrid algorithms, we identified 5550 of their target genes. Further analyses with the Gene Ontology term and KEGG pathways for these predicted target genes suggested that the miRNAs may be involved in key developmental biological processes. Our results provide the first repository of *G. pallidipes* miRNAs across developmental stages, some of which appear to play crucial roles in tsetse fly development. Hence, our findings provide a better understanding of tsetse biology and a baseline for exploring miRNA genes in tsetse flies.

**Availability and implementation:**

Raw sequence data are available from NCBI Sequence Read Archives (SRA) under Bioproject accession number PRJNA590626.

**Supplementary information:**

Supplementary data are available at *Bioinformatics Advances* online.

## 1 Introduction

African animal trypanosomosis (AAT) caused by the parasites *Trypanosoma vivax*, *Trypanosoma brucei brucei* and *Trypanosoma congolense* and their tsetse fly vector, *Glossina pallidipes* (Diptera: Glossinidae), is an essential constraint to livestock production and a threat to food security in sub-Saharan Africa (SSA). Up to 20% revenue losses have been estimated due to trypanosome infections in cattle that result in mortality and reduced calving rates and milk and meat production. The tsetse trypanosome burden also indirectly affects crop production by reducing draft animals and production of manure for fertilizer (Holt *et al.*, 2016; Saini *et al.*, 2017). Hence, the most effective way of managing the burden is by tsetse control such as with odor-baited traps or repellents, insecticide-treated targets and sterile insect technique (Saini *et al.*, 2017; Vreysen *et al.*, 2013). A better understanding of the genetic regulatory mechanisms involved in *G.pallidipes* development and metamorphosis can help identify new vector control approaches.

MicroRNAs (miRNAs) are small non-coding RNA of 18–25 nucleotides (Lucas and Raikhel, 2013). Several studies suggest that miRNAs regulate gene expression in almost all physiological and biological processes in both eukaryotes (Carthew and Sontheimer, 2009). The main public repository and online resource for miRNA sequences, miRBase, have about 48 860 mature miRNA sequences and annotation for 271 organisms (Kozomara *et al.*, 2019). However, only 11% of these mature miRNA genes are from different insect species, despite their regulatory importance in processes such as organ development and metamorphosis (Caygill and Johnston, 2008), reproduction (Perry *et al.*, 2014), behaviour and neurogenesis, as well as host–pathogen interactions (Asgari, 2013; Meki *et al.*, 2018). To understand the function of miRNAs during metamorphosis of any given organism, it is important to know the temporal expression profiles of these genes across their developmental cycles (Allam *et al.*, 2016; Jain *et al.*, 2015). Currently, the most efficient way to determine a comprehensive coverage of miRNA profiles in non-model species is by using next-generation sequencing approaches. Some studies have successfully employed this technique to identify miRNA genes, even when well-annotated genomes are lacking (Calla and Geib, 2015; Feng *et al.*, 2018a).

In a previous study, miRNAs were identified in *G.pallidipes* as regulating different processes when infected with the salivary gland hypertrophy virus (Meki *et al.*, 2018). However, how miRNAs are regulated across *G.pallidipes* developmental stages remains unknown. To generate baseline miRNA profiles of *G.pallidipes* for subsequent investigations, such as developmental processes and their role in pathogen transmission, we sequenced small RNA (sRNA) libraries from both sexes of larval, pupal, teneral adult and non-teneral adult developmental stages and from gravid female adults. We then used bioinformatics approaches to investigate the miRNAs and their functional groups. The repository of the miRNA generated from this study may provide knowledge on tsetse biology and facilitate the development of new vector control strategies.

## 2 Methods

### 2.1 Insect rearing

The specimens compared in this study included three replicates each of *G.pallidipes* larvae, pupae, teneral males and females, non-teneral males and females (unmated) and gravid females. *Glossina pallidipes* were reared in the insectary at the International Centre of Insect Physiology and Ecology (*icipe*) under favourable conditions of 75–90% humidity and 23°C ± 1°C. The adults were fed once on defibrinated bovine blood on an *in vitro* membrane feeding system and then starved 2 days before RNA extraction. The third instar larvae were collected immediately after larviposition and frozen at −80°C to prevent the larvae from transitioning to pupae. Based on other studies, we believe that freezing the larvae at −80°C did not significantly affect the integrity of our RNA and the sRNA expression pattern (Hernandez *et al.*, 2009; Park *et al.*, 2013, 2017; Seelenfreund *et al.*, 2014; Xiao *et al.*, 2015). Other larvae were left for 2 days, which were then collected as 2-day-old pupae. Newly emerged flies (teneral flies) and mature 8-day-old adult flies (non-teneral flies) of both sexes were also captured from the insectary. Whole bodies of all specimens were immobilized at 4°C for 10 min before total RNA extraction.

### 2.2 Female tsetse fly dissection

The gravid females carrying fully developed third instar larvae were observed visually and selected for dissection. They were immobilized by placing them in the refrigerator at 4°C for 10 min. Each uterus was dissected under a microscope, and the larvae were carefully removed to maintain the integrity of the uterus of the female specimens (Attardo *et al.*, 2014). The carcasses of the gravid females were used for total RNA extraction.

### 2.3 RNA isolation and sRNA library preparation

All samples were prepared in three biological replicates; total RNA was extracted using Trizol^®^ Reagent per the manufacturer’s instructions. The quality of the total RNA was measured using Eppendorf Biospectrometer^®^ (Hamburg, Germany) by the ratio of OD260 and OD280; the value of all samples ranged from 1.8 to 2.2, indicating good quality of the RNA. The Agilent 2100 Bioanalyzer further determined the quality according to the manufacturer’s instructions. Total RNA with RNA integrity numbers (RINs) of 7.5–8.9 were used to prepare non-coding sRNA. The sRNA fragments of <40 bp were separated on polyacrylamide gels. Subsequently, the sRNA fragments were ligated with 3ʹ RNA adaptors and used to prepare libraries that were sequenced using Illumina sequencing platform Hiseq 2500 (Macrogen, South Korea). Raw sequencing data were submitted to NCBI Sequence Read Archives (SRA) under Bioproject accession number PRJNA590626.

### 2.4 Data analysis

#### 2.4.1 Read quality check and assessment

The 21 sRNA libraries were checked for quality using fast QC software. An in-house script (https://github.com/CareenNaitore/Snakemake_project/blob/master/script.folder/adapter_removal.sh) was used to remove low-quality sequences, contaminants and adaptors of 70-bp length using the Cutadapt software (v1.18; Martin, 2011). The 21 sequence libraries were rechecked for quality by the FastQC software (v0.11.4; https://www.bioinformatics.babraham.ac.uk/projects/fastqc/) for post-sequencing data analysis.

#### 2.4.2 Small non-coding RNA annotation

The small non-coding RNA in the *G.pallidipes* reference genome (GenBank: KK499764) were structurally predicted using the cmscan algorithm from the Infernal software packages v1.1.2 (Nawrocki and Eddy, 2013) against the RNA families (RFAM) database v.14.0 (Nawrocki *et al.*, 2015). Once small non-coding RFAM were identified in the genome, the clean reads of 18–35 bp from the 21 different libraries were aligned using Bowtie (v1.2.1.1), retaining the sequences with only one mismatch at the seed region and the reads that do not align to more than five different locations in the genome (Allam *et al.*, 2016). Finally, we used BEDTools (v2.27.1; Quinlan and Hall, 2010) to determine the overlapping small non-coding RNAs in the sequence libraries allowing for mismatches of only one nucleotide.

#### 2.4.3 MiRNA sequence identification

The miRNA genes were identified using miRDeep2 (v2.0.0.8) software (Friedländer *et al.*, 2012) and insect miRNA sequences from miRBase database, release 22 (Kozomara *et al.*, 2019). The mapper module from miRDeep2 aligns the pre-miRNA from miRBase database to the *G.pallidipes* genome, ensuring that there is a match at the star region, loop region and mature region of the candidate. Once the pre-miRNAs candidates are identified and extracted from the reference genome, they are folded to hairpin structures, and the folding energy of the precursor is determined using an algorithm from the randfold software (v 2.0.1; Friedländer *et al.*, 2012) and ViennaRNA packages (v 2.3.5; Lorenz *et al.*, 2011). The module *miRDeep2.pl* was used to identify the miRNA genes in our libraries. The information provided was further filtered by examining miRDeep2 score of the hairpin structures (Friedländer *et al.*, 2012) and if the miRNA genes were represented in more than one sample replicate and had a score of <2 was assigned an identifier from miRBase release 22 (Kozomara *et al.*, 2019). The sRNA sequences that were not provided with identification from miRBase were further validated by the algorithm parameters set in miRDeep2 (v 2.0.08), a minimum folding energy of <−20 kcal/mol, identification of a dicer and a drosha cleavage and miRDeep2 score of ≥2 (Ambros *et al.*, 2003). Consequently, the parameters were used as the cut-off level to declare them as potential novel miRNA candidates.

#### 2.4.4 MiRNA differential expression analysis

Read counts generated from the quantifier script in miRDeep2 were used for statistical analysis by Perseus software (v1.6.2.3; Tyanova *et al.*, 2016). The read counts were first log2 transformed and Pearson correlation coefficients calculated within the three biological replicates in each life stage. However, we had to exclude one replicate from the larval samples due to low Pearson correlation (*R* < 0.70) among the other replicates. Subsequently, we identified differentially expressed genes using edgeR (Empirical Analysis of Digital Gene Expression Data in R; v3.16.5) package (Robinson *et al.*, 2010). The normalized mean values of the miRNA genes were used to determine their abundance in each life stage. The normalized values were in the form of counts per millions of the total number of reads (CPM), miRNA with less than 10 CPM were filtered from the analysis. The miRNA with a false discovery rate adjusted *P*-value of ≤0.05 and the fold change of log2 scale of >1 or <−1 were determined as either upregulated or downregulated in subsequent life stages.

#### 2.4.5 MiRNA target prediction and functional analysis

The potential candidate target genes for the differentially expressed miRNAs were identified by RNAhybrid v2.1.2 (Krüger and Rehmsmeier, 2006) and miRanda v3.3a (http://www.microrna.org/microrna/home.do; Betel *et al.*, 2010; Lucas and Raikhel, 2013). Orthologous genes of *Glossina morsitans morsitans* 3ʹUTR sequences obtained from VectorBase (www.vectorbase.org/) were used to identify potential miRNA gene targets in *G.pallidipes*. RNAhybrid software v2.1.2 was set with the parameters: miRNA:mRNA binding energy <−14 kcal/mol, the *P*-value was set at 0.05 and >6 nucleotide match at the seed region (Krüger and Rehmsmeier, 2006). MiRanda software (v 3.3a) was used with the parameters: miRNA:mRNA binding energy value <−14 kcal mol and a score of ≥100. The potential candidates identified by both softwares were selected for further analysis. The Gene Ontology (GO) and pathway annotation of the selected candidate genes was performed using Blast2GO version 5.2.5 (Conesa *et al.*, 2005).

## 3 Results

### 3.1 sRNA distribution

We obtained about 149 478 030 raw reads (Table 1) of up to 101 nt in length from the 21 sequence libraries. After cleaning the reads through the removal of adapters, 139 538 937 reads remained. The quality of libraries was further improved by removing contaminated reads such as 2S rRNA (5ʹ-TGCTTGGACTACATATGGTTGAGGGTTGTA-3ʹ) and 5.8S rRNA (5ʹ-CTGCTTGGACTACATATGGTTGAGGGTTGTA-3ʹ) and low-quality sequences to remain with 50 460 899 clean reads (Table 1). The clean reads across all 21 libraries had most of the sequences distributed from 17 to 35 nt (Supplementary Fig. S1), the standard size of sRNAs genes (Allam *et al.*, 2016; Feng *et al.*, 2018a). However, low percentages of the clean reads mapped to the *G.pallidipes* genome; from larval, pupal, teneral female, teneral male, non-teneral female, non-teneral male and gravid female libraries, 38.54%, 30.96%, 38.04%, 29.46%, 47.33%, 34.50% and 56.70% mapped to *G.pallidipes*, respectively (Table 1). We were nonetheless able to categorize the reads into distinct RNA genes. The relative abundances of sRNA distributions in the life stages are shown in Supplementary Figure S2, with high abundance in tRNA, miRNA and rRNA genes.

**Table 1.** Summary of sRNA sequencing data analysis for the five life-stage libraries of *G. pallidipes*

Stage	Sex	Replicates	Total raw counts	Reads post-adaptor trimming	High-quality reads (17–35)	Mapped to the genome	Unique reads
Larvae		3	20 361 758	19 691 042 (96.71%)	5 652 804 (28.71%)	2 178 368 (38.54%)	17 048
Pupae		3	23 201 822	20 262 789 (87.33%)	8 128 822 (40.60%)	2 516 749 (30.96%)	14 455
Teneral adults	Female	3	20 989 380	20 019 810 (95.38%)	6 119 010 (30.56%)	2 327 379 (38.04%)	42 881
	Male	3	20 914 152	20 877 543 (99.82%)	5 197 405 (24.89%)	1 531 138 (29.46%)	31 916
Non-teneral adults	Female	3	21 971 711	19 918 765 (90.66%)	8 879 712 (44.58%)	4 203 245 (47.33%)	40 964
	Male	3	19 771 800	19 058 192 (96.91%)	7 184 851 (37.70%)	2 478 727 (34.50%)	52 541
Gravid	Female	3	22 267 407	19 710 796 (88.52%)	9 298 295 (47.17%)	5 271 811 (56.70%)	57 529
Total		21	149 478 030	139 538 937 (93.35%)	50 460 899 (36.16%)	20 507 417 (40.60%)	257 334

### 3.2 MiRNA identification and annotation

**Fig. 1.**
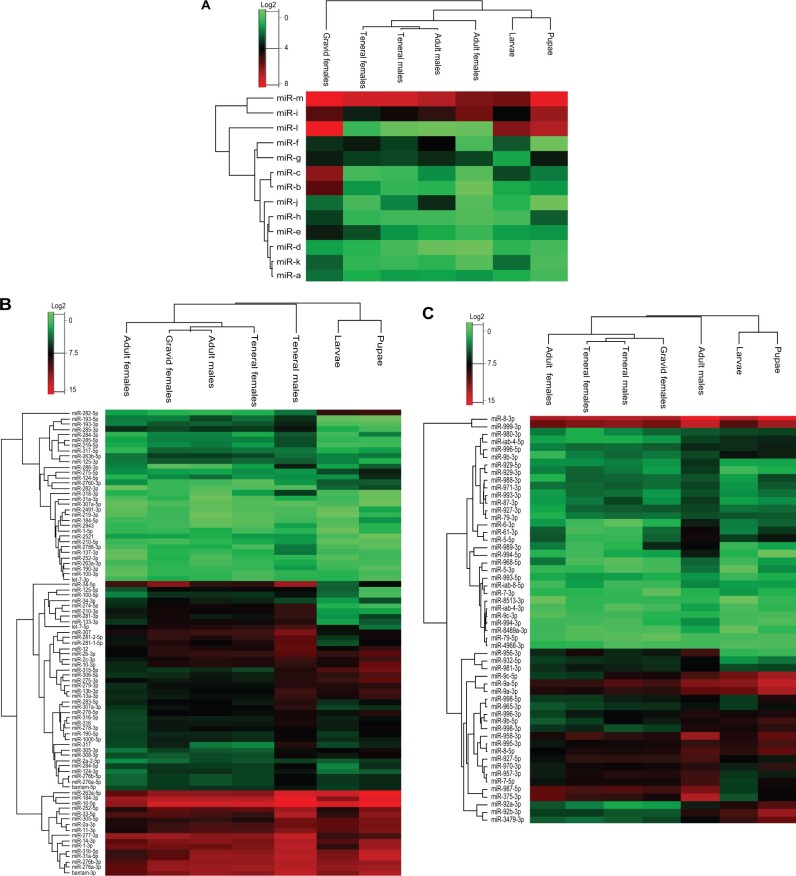
Heatmaps for clustering of *G. pallidipes* miRNAs. The heatmaps represents the log2 transformation of the read counts from (**A**) novel miRNA genes and (**B** and **C**) 92 and 53 known miRNA genes, respectively. The colour gradually increases from the light green colour (low expression) to dark red colour (high expression), indicating the relative expression profiles of miRNA in the respective life stages

During our analysis, the sRNA libraries from the developmental stages (larvae, pupae, teneral adults, non-teneral adults and gravid females) produced more than 3 million reads coding for miRNA genes in *G.pallidipes* (Table 1). We characterized a total of 157 miRNA genes (Supplementary Table S1), out of which we identified 148 genes in larvae, 137 in pupae, 144 in teneral female adults, 139 in teneral male adults, 137 in non-teneral adult males, 156 in non-teneral adult females and 141 in gravid adult females. These included 99 miRNA genes already computationally predicted in a recent study by Meki *et al.* (2018) and annotated in the *G.pallidipes* genome hosted by VectorBase (Megy *et al.*, 2012). We also identified a total of 47 mature miRNA gene candidates as orthologs of other insects. However, 8 of the 47 miRNA genes (miR-375-3p, miR-7-5p, miR-957-3p, miR-970-3p, miR-980-3p, miR-993-3p, miR-306-3p and mir-307-3p) were conserved in more than one insect species (*Drosophila melanogaster*, *Anopheles gambiae*, *Aedes aegypti*, *Apis melifera* and *Bombyx mori*) catalogued in miRBase. After identifying the conserved miRNA genes, we predicted 12 novel candidates from the *G.pallidipes* genome. Interestingly, the miRDeep2 results of these novel candidate genes showed no conserved seed region to any known families in miRBase. Hence, the genes were named in a particular sequence, as shown in Figure 1A.

### 3.3 Most abundant miRNA sequences in the different developmental stages

The most abundant known miRNAs identified included miR-8-3p (357 999 reads), miR-184-3p (353 418 reads), miR-10-5p (523 285 reads), miR-263a-5p (288 610 reads), miR-277-3p (147 188 reads) miR-276a-3p (149 681 reads), miR-276b-3p (119 331 reads), bantam-3p (111 453 reads), miR-31a-5p (113 648 reads) and miR-1-3p (95 462 reads; Fig. 1B and C). Among these, only miR-1-3p, miR-8-3p and miR-184-3p were previously identified as the abundant miRNA genes in *G.pallidipes* (Meki *et al.*, 2018). Interestingly, four of the novel miRNAs, miR-b (209 reads), miR-c (487 reads), miR-h (100 reads) and miR-m (2647 reads), were highly enriched only in non-teneral females. In contrast, miR-l was highly expressed with more than 500 reads not only in non-teneral females, but also in gravid females, larvae and pupae (Fig. 1A).

### 3.4 Sex-specific expression profiles

**Fig. 2.**
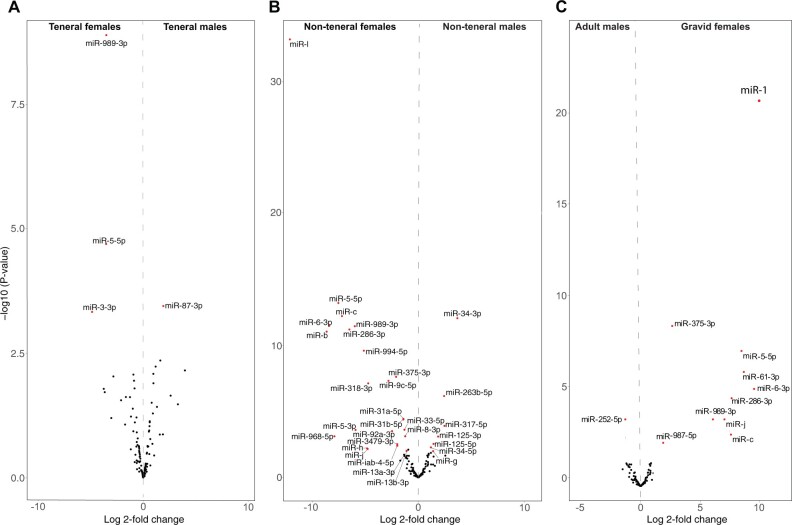
Volcano plots of differentially regulated sex-specific miRNAs in *G. pallidipes*. miRNA expression differences [log2(fold change), *x* axis] and their statistical significance (−log10 *P*-values, *y* axis) between the sexes among (**A**) teneral, (**B**) non-teneral flies and (**C**) adult male versus gravid female *G. pallidipes* are plotted. Red dots represent significantly upregulated (*α* = 0.05; −1 > fold change > +1) miRNA genes in different sexes

Three miRNAs (miR-5-5p, miR-989-3p and miR-3-3p) were upregulated and one miRNA (miR-87-3p) was downregulated in newly emerged teneral female adult flies compared to teneral male adults as shown in Figure 2A. We identified 24 miRNAs that were more highly expressed in the non-teneral adult females than in the non-teneral adult males (Fig. 2B). Seven miRNAs (miR-133-3p, miR-125-5p, miR-125-3p, miR-34-5p, miR-34-3p, miR-317-5p, miR-263b-5p and miR-g) were significantly downregulated in the non-teneral females compared to the non-teneral males (Fig. 2B). Similarly, one miRNA (miR-252-5p) was significantly downregulated in gravid females compared to adult males. However, 10 miRNAs were significantly upregulated in gravid females compared to non-teneral adult males (Fig. 2C).

### 3.5 MiRNA regulation during tsetse development

**Fig. 3.**
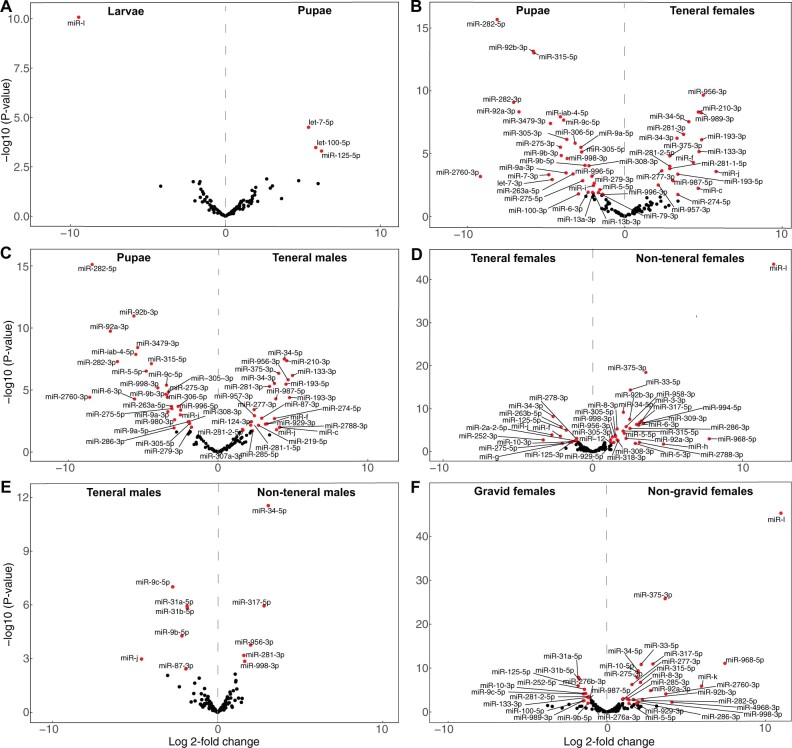
Volcano plots of differentially regulated stage-specific miRNAs in *G. pallidipes*. MiRNA expression differences [log2(fold change), *x* axis] and their statistical significance (−log 10 *P*-values, *y* axis] between the consecutive *G. pallidipes* life stages are plotted; (**A**) larvae versus pupae, (**B**) pupae versus teneral females, (**C**) pupae versus teneral males, (**D**) teneral versus non-teneral females, (**E**) teneral versus non-teneral males and (**F**) gravid versus non-gravid (teneral) females. Red dots represent significantly upregulated (*α* = 0.05; −1 > fold change > +1) miRNA genes in the different life stages

The pupation process revealed only four differentially expressed miRNAs; three miRNAs (let-7-5p, miR-100-5p and miR-125-5p) were upregulated in the pupal stage (Fig. 3A), and one novel miRNA (miR-i) was downregulated in the pupal stage compared to the larval stage. Metamorphosis of pupae to teneral female adults was associated with the most significant shift in miRNA expression; we identified 18 miRNA that were highly expressed in the teneral female stage compared to the pupal stage and 32 miRNA genes that were downregulated in the teneral female stage compared to the pupal stage (Fig. 3B). The miRNA miR-989-3p was upregulated in the teneral female specimen compared to the pupae, which indicates the importance of this miRNA in the female metamorphosis. In addition, we observed upregulation of 24 miRNAs and downregulation of 28 miRNA in the teneral male adults compared to pupae (Fig. 3C). The transition from newly emerged (teneral) into mature (non-teneral) flies was associated with 41 miRNA genes significantly differentially expressed, 29 miRNA genes were upregulated in non-teneral stage and 12 miRNA genes were downregulated in the non-teneral female stage (Fig. 3D). In contrast, 11 miRNA genes were differentially expressed between the transition of teneral male adults and non-teneral male adults; five were upregulated and six were downregulated in non-teneral male compared to the teneral male flies (Fig. 3E). We identified 25 miRNA genes that were significantly upregulated and 18 miRNA genes that were downregulated in the gravid females compared to non-gravid (teneral) females (Fig. 3F).

### 3.6 Target prediction and functional annotation

**Fig. 4.**
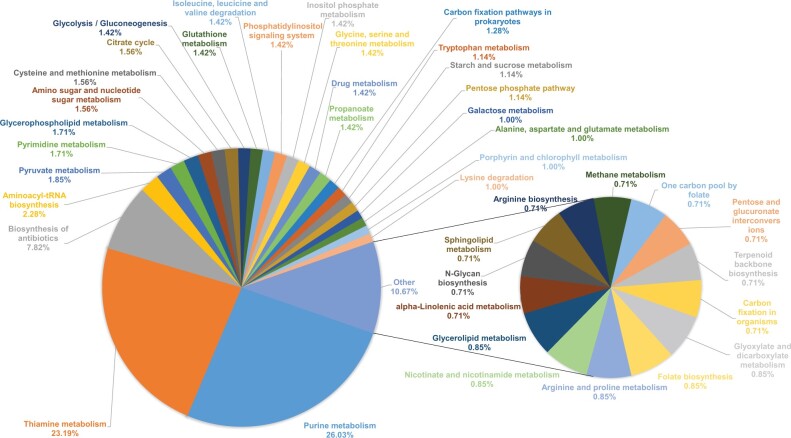
Pie charts of KEGG pathway predicted target genes of *G. pallidipes* miRNA. Various KEGG pathways with > and <1.00% of miRNA target genes are represented in separate pie charts

**Fig. 5.**
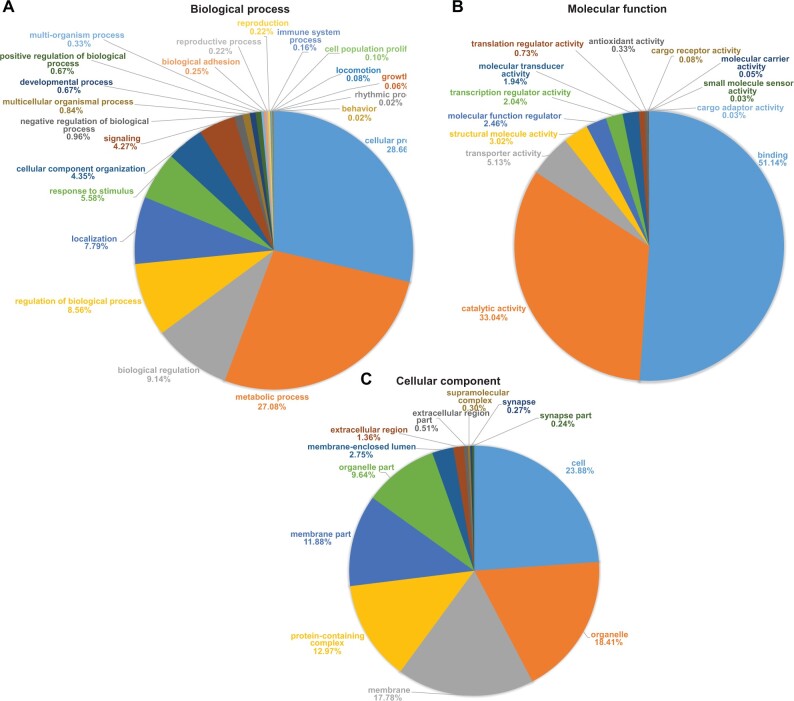
Pie charts of GO classifications. Classifications of 5550 putative target genes of *G. pallidipes* miRNA involved in (**A**) biological process, (**B**) molecular function and (**C**) cellular component

Considering only differentially expressed miRNA genes in the various developmental stages, 5550 putative miRNA–mRNA pairs were predicted by RNAhybrid and miRanda. The transcript genes that overlapped were further analysed using Blast2GO, which yielded KEGG pathways enriched in purine metabolism (*n* = 183), thiamine metabolism (*n* = 163), biosynthesis of antibiotics (*n* = 53), aminoacyl-tRNA biosynthesis (*n* = 16), pyruvate metabolism (*n* = 13; Fig. 4). In addition, functional GO terms involved in biological processes were enriched as cellular process (*n* = 1464), metabolic process (*n* = 1383), biological regulation (*n* = 467), localization (*n* = 328), as well as cellular component enriched in the cell (*n* = 1313) and organelle components (*n* = 1012) and molecular function enriched in binding process (*n* = 2812) and catalytic activity (*n* = 1817; Fig. 5) were also predicted.

We generated eight miRNA–mRNA networks to demonstrate their interaction at different stages of development and sexes. The number of targets for each network varied depending on the number of miRNA genes (Supplementary Figs S3 and S4). We found out that the miRNA–mRNA network of larvae versus pupae had four differentially expressed genes targeting 159 mRNA transcript targets (Supplementary Fig. S3A). The miRNA–mRNA network of pupae versus teneral females had 50 differentially expressed miRNA genes targeting 166 mRNA transcripts (Supplementary Fig. S3B). The miRNA–mRNA network of pupae versus teneral male adults had 51 differentially expressed miRNA genes targeting 305 mRNA transcripts target (Supplementary Fig. S3C). The miRNA–mRNA network of teneral versus non-teneral females had 41 differentially expressed miRNA genes targeting 637 mRNA transcripts (Supplementary Fig. S3D). The miRNA–mRNA network of teneral versus non-teneral males identified 127 mRNA gene transcripts targeted by 11 differentially expressed miRNAs in Supplementary Figure S3F. The miRNA–mRNA network of gravid versus non-gravid (teneral) females identified 273 mRNA gene transcripts targeted by 43 differentially expressed miRNAs (Supplementary Fig. S3E). In addition, we demonstrated the sex-specific miRNA–mRNA network interactions for the adult stages (teneral and non-teneral). The miRNA–mRNA network of teneral males versus teneral females had four differentially expressed miRNA genes targeting 55 mRNA gene transcripts (Supplementary Fig. S4A). Also, the miRNA–mRNA network of non-teneral males versus non-teneral females had 30 differentially expressed miRNA genes targeting 234 mRNA transcript genes (Supplementary Fig. S4B). These networks also indicated that several miRNAs had *in silico* binding sites for more than one mRNA gene. For instance, miR-34-5p, miR-125-5p, miR-100-5p and miR-275-3p had 7, 13, 19 and 11 mRNA binding sites, respectively (Supplementary Figs S3 and S4), indicating that the miRNA genes may have potential in regulating more than one mRNA transcript in the species.

## 4 Discussion

In this study, the miRNA of *G.pallidipes* was profiled using next-generation sequencing technology. The libraries obtained from the life stages of the different sexes included over 50 million high-quality reads. However, the reads from libraries aligned to the *G.pallidipes* genome at low percentages ranging from 38.54% to 56.70%, which could be attributed to the short nature of the reads and the high complexity of the unmasked reference genome as observed in other studies (Allam *et al.*, 2016; Calla and Geib, 2015). Despite the low mapping quality, sRNA genes were characterized in the various *G.pallidipes* developmental stages (larvae, pupae, male and female teneral adults, male and female non-teneral adults and gravid females). The largest classes of sRNA genes characterized included miRNA, tRNA and rRNA, as also observed in *Anopheles funestus* (Allam *et al.*, 2016). Meki *et al.* (2018) computationally classified 57 miRNA genes in *G.pallidipes* infected with salivary gland hypertrophy virus. However, we focused on the developmental stages of *G.pallidipes*, identifying 157 mature miRNAs genes, among which 12 were characterized as novel miRNA. The miRNA genes with the highest number of reads in all developmental stages included miR-10, miR-8, miR-184 and miR-263a, similar to in other insects such as *An.gambiae* (Biryukova *et al.*, 2014) and *Grapholita molesta* (Wang *et al.*, 2017).

MiRNA clusters play a crucial role in evolutionary conservation among eukaryotes and most are clustered in tandem repeats in their genome (Axtell *et al.*, 2011). Recently, differences between male and female miRNA expression profiles have been observed in various insect species (Marco, 2014), suggesting their role in sex determination or sex-specific functions. In the assembled genome of *G.pallidipes* (GenBank: KK499764), we identified female-biased clusters (miR-309-3p/miR-3-3p/miR-286-5p/miR-5-5p/miR-5-3p/miR-6-3p, miR-318-5p/miR-994-5p, miR-92a-3p/miR-92b-3p/miR-3479-3p and miR-375-3p/miR-375-5p) and male-biased clusters (miR-125-5p/miR-125-3p, miR-34-5p/miR-34-3p). The pan-drosophilid miR 309∼6 cluster of members (miR-309, miR-3, miR-286, miR-5, miR-6) were identified to align in a region of 4.31 kb in scaffold 26 of the *G.pallidipes* genome assembly (GenBank: KK499764). The same cluster has been observed in other insects, and has been hypothetically attributed to duplication, insertion or deletion of members of the cluster (Marco, 2014).

This study is the first report on the developmental regulation of miRNA across metamorphosis in tsetse flies. The miRNA cluster let-7-complex locus (let-7-5p, miR-100-5p and miR-125-5p) is highly conserved among animal species, especially insects. In *D.melanogaster*, the let-7 complex is a spatial–temporal gene that is highly expressed in the pupal and adult stages (Caygill and Johnston, 2008) and regulates the temporal identity of *Drosophila* mushroom body neurons via chinmo genes (Wu *et al.*, 2012). In *G.pallidipes*, we identified these orthologous genes clustered in a 1.284-kb interval in scaffold 60 with significant expression during the transition of larvae to pupae, indicating its role in metamorphosis of the insect.

In *G.pallidipes*, sexual dimorphism cannot be easily observed in the early developmental stages (larvae to pupae) as demonstrated in other holometabolous insects such as *D.melanogaster* (Perry *et al.*, 2014), *Musca domestica* (Dübendorfer *et al.*, 2002) and *Stomoxys calcitrans* (Dowell *et al.*, 2005). Hence, our identification of stage-specific miRNA expression across metamorphosis was between unsexed larvae and pupae and in teneral adults of both sexes. The metamorphosis process involves organ and tissue reorganization, which requires a lot of molecular interactions as demonstrated with the significant number of miRNAs being expressed. Among these, miR-iab-4-5p, miR-193-3p/miR-193-5p, miR-210-3p and miR-281-3p/miR-281-1-5p/miR-281-2-5p showed significant expression in adult stages (teneral) compared to the pupal stage. miR-193 and miR-210 were also similarly expressed in *Anopheles sinensis* (Jain *et al.*, 2015). Previous studies have demonstrated that miR-iab-4-5p and miR-193 have crucial roles in post-pupation stage development in other Diptera species such as *Heliconius melpomene* (Surridge *et al.*, 2011) and *Drosophila* (Cusumano *et al.*, 2018; Rosenhaugen *et al.*, 2005). Nevertheless, sex-biased miRNA such as miR-989-3p and miR-87-3p had different expression patterns between male and female metamorphosis, indicating their important roles during the transition from immature (pupa) to mature (teneral adult) stages, as found in *D.melanogaster* and *Anopheles* spp. (Allam *et al.*, 2016; Feng *et al.*, 2018b; Jain *et al.*, 2015; Kugler *et al.*, 2013). Interestingly, miR-282-5p, which was highly expressed in the *G.pallidipes* pupal stage compared to the adult teneral stage [*P* < 0.0001 and |log2 (fold change)| > 1], had similar expression patterns in both *D.melanogaster* and *Bactrocera dorsalis* (Calla and Geib, 2015). This miRNA has been shown to regulate viability and production of eggs through the targeting of the nervous-specific adenylate cyclase in pupae during metamorphosis (Vilmos *et al.*, 2013).

When teneral flies take up a blood meal and use the nutrients for body maturation, several changes occur, especially in gene expression (Haines, 2013) that influence its morphology when transitioning into a mature adult (non-teneral adults). The morphological differences play a crucial role in vector competence and reproduction (Haines, 2013). The transformation from teneral to non-teneral adults is tightly regulated by a multitude of expressed miRNAs. In our study, miR-317-5p, miR-34-5p, miR-956-3p and miR-958-3p were upregulated in non-teneral (male and female) compared to teneral (male and female) flies [*P* < 0.001 and |log2 (fold change)| > 1]. Interestingly, these clusters have been shown to play crucial roles in insect immune development. For instance, miR-956 has been linked to inhibited viral replication in *Drosophila* through downregulation of the ectoderm-expressed 4 (Ect4) gene (Monsanto-Hearne *et al.*, 2017), and miR-958 has appeared to inhibit Toll signalling and drosomycin expression by direct targeting of *Toll* and *Diff*, which are involved in the innate immune response of *D.melanogaster* (Li *et al.*, 2017).

Some of the miRNAs have demonstrated vector competence functions, such as miR-34, which has been shown to be highly expressed during *Plasmodium* parasite invasion in *An.gambiae*, indicating its role in mosquito vector competence (Biryukova *et al.*, 2014). The miRNA, miR-275 has been linked to tsetse vector competence and is postulated to regulate the synthesis of the *proventricular membrane* by modulating the Wnt signalling pathway and the iroquois (iro/Irx) family of transcription factors in *G.morsitans* (Aksoy *et al.*, 2016). However, the ortholog gene in *Ae.aegypti* females has been shown to regulate blood meal-activated physiological events involved in the complete maturation of the egg (Bryant *et al.*, 2010). In our study, miR-275 was upregulated in the non-teneral female stage compared to the teneral female stage, indicating that the gene might be involved in immune responses and functions related to reproduction.

Adult female tsetse undergoes viviparous reproduction—generating one larva each gonotrophic cycle throughout its lifetime—an investment that promotes offspring survival (Attardo *et al.*, 2012). The uterus has to expand 100 times to fit the growing larvae (Benoit *et al.*, 2017), and lipid reservoirs from their fat bodies are metabolized for larval nourishment. We identified miR-277, which was among the highly expressed miRNA genes in the gravid females and was also more abundant than in non-gravid (teneral) females. In *Ae.aegypti* mosquitoes, miR-277 targets *insulin-like peptides 7* and *8* to control reproduction and lipid metabolism (Ling *et al.*, 2017). MiRNA genes have been shown to control post-translational initiation and mRNA stability in eukaryotic species. We wanted to understand the regulatory functions of the various miRNA genes by identifying the target genes and evaluating their putative roles.

MiRNA genes are known to bind to the 3ʹUTR of the mRNA genes (Lucas and Raikhel, 2013). However, recent studies have shown that miRNA genes can also bind to 5ʹUTR and coding regions of the mRNA, causing either their degradation or translation inhibition (Gu *et al.*, 2014; Forman *et al.*, 2008; Kloosterman *et al.*, 2004). In our study, we mainly focused on identifying the target genes sites using two algorithms (miRanda and RNAhybrid) with the reference genome of *G.morsitans morsitans* as it is well-annotated compared to *G.pallidipes* genome. Other studies have exploited similar avenues to reduce limitations of genome annotation in identifying putative target genes (Feng *et al.*, 2018a, b; Rebijith *et al.*, 2016; Wang *et al.*, 2017). The differentially expressed miRNAs were used to identify 5550 mRNA transcripts that have potential target site for our miRNA genes, with numerous biological functions and pathways. During the metamorphosis of larvae to non-teneral adults, we found that biological processes such as cellular process, metabolic process, organelle, membrane, cellular component organization or biogenesis and enriched pathways such as purine metabolism, thiamine metabolism and aminoacyl-tRNA biosynthesis were among the predicted functional roles of the miRNA target gene list. These biological processes and key pathways might be involved in the formation and regeneration of tissues in the various life stages of *G.pallidipes*.

Networks of the miRNA and mRNA that were visualized were further analysed and the miRNA genes were predicted to target at least one mRNA target site each. We predicted the biological roles of the developmental miRNA genes in the different stages and sexes of tsetse. For example, GMOY009801, an orthologous gene of GPAI002137, which is targeted by more than one miRNA, is an annotated gene (TSC2 complex) in many insects, including *G.pallidipes* (Liu *et al.*, 2009), and plays a vital role in negative regulation of the mammalian target of the rapamycin (mTOR) signalling pathway (Dubinsky *et al.*, 2014). The correlation between miRNA genes and its target genes may reflect a regulatory mechanism used by the insect to alter biological processes for its development. However, target genes are limited to computational prediction (Chaudhuri and Chatterjee, 2007). Therefore, experimental studies such as miRNA luciferase knock-down are required to verify the predicted genes (Jin *et al.*, 2013).

In conclusion, our study provides a comprehensive baseline account of the miRNA expression patterns across the five different life stages and sexes of *G.pallidipes*. Most miRNAs showed stage- and sex-specific expression patterns. For example, miR-989, which was significantly higher expressed in the adult females compared to adult males, could be further probed to enhance understanding of its function. Further empirical confirmations of the miRNA target genes will provide a better understanding of their biological function in tsetse biology and their potential utility in the development of new effective tsetse control tools.

## Supplementary Material

vbab047_Supplementary_Data
