# Supplementary material for: The developmentally dynamic microRNA transcriptome of Glossina pallidipes tsetse flies, vectors of animal trypanosomiasis
Source: Bioinform Adv. 2021 Dec 28;2(1):vbab047. doi: 10.1093/bioadv/vbab047 (PMC9710702; doi:10.1093/bioadv/vbab047)
Supplement: vbab047_Supplementary_Data [file vbab047_supplementary_data.zip › Supplementary figure 1.docx]

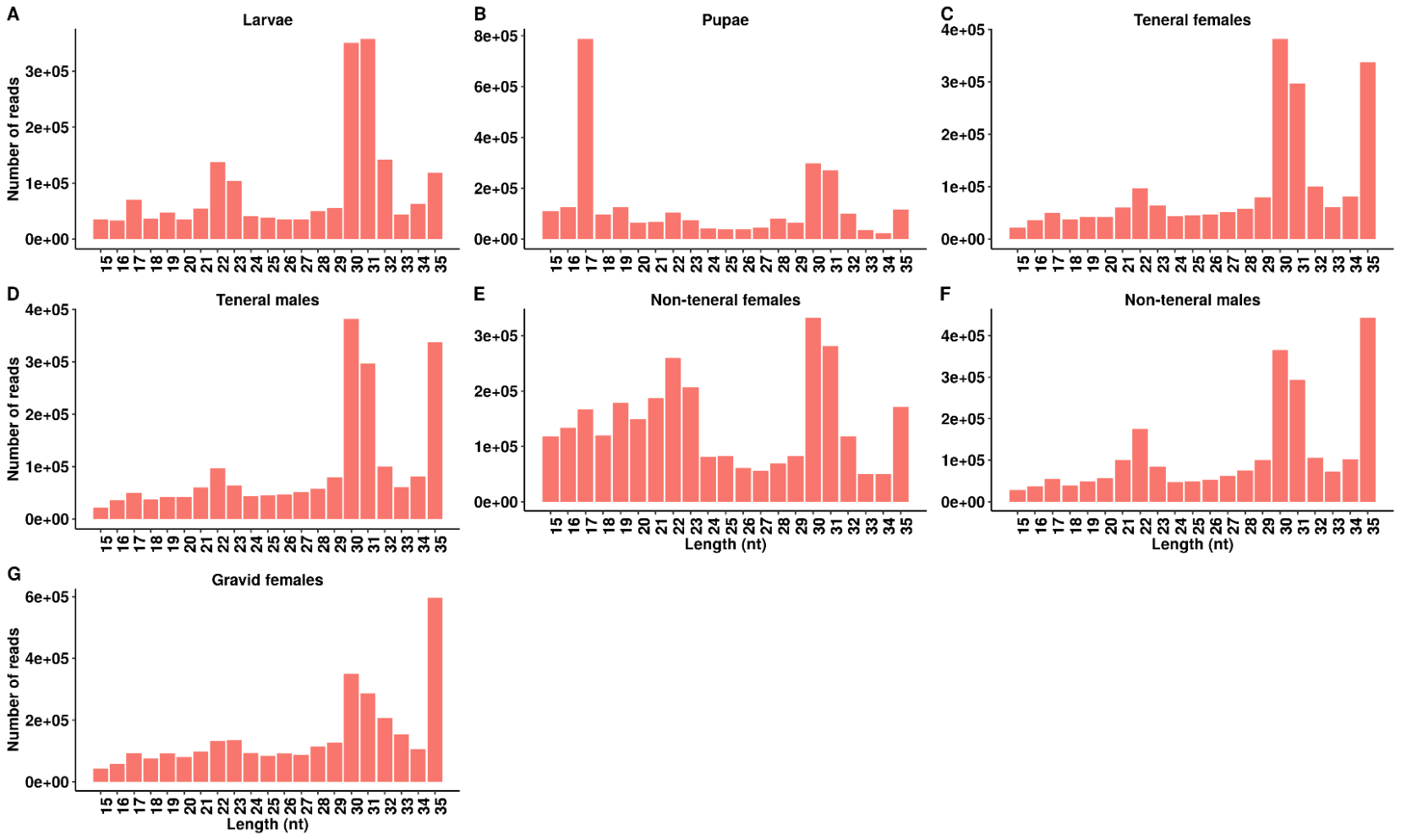


**Supplementary figure 1:** The length distribution of *G. pallidipes* small RNA sequences pooled in (A) larvae libraries, (B) pupae libraries, (C) teneral females libraries, (D) teneral males libraries, (E) non-teneral females libraries, (F) non-teneral males libraries, and (G) gravid females libraries. The y-axes represent the absolute number of reads (x 100,000) in each library and the x-axes represent the length of the reads in nucleotides (nt).
