# Supplementary material for: The developmentally dynamic microRNA transcriptome of Glossina pallidipes tsetse flies, vectors of animal trypanosomiasis
Source: Bioinform Adv. 2021 Dec 28;2(1):vbab047. doi: 10.1093/bioadv/vbab047 (PMC9710702; doi:10.1093/bioadv/vbab047)
Supplement: vbab047_Supplementary_Data [file vbab047_supplementary_data.zip › Supplementary figure 2.docx]

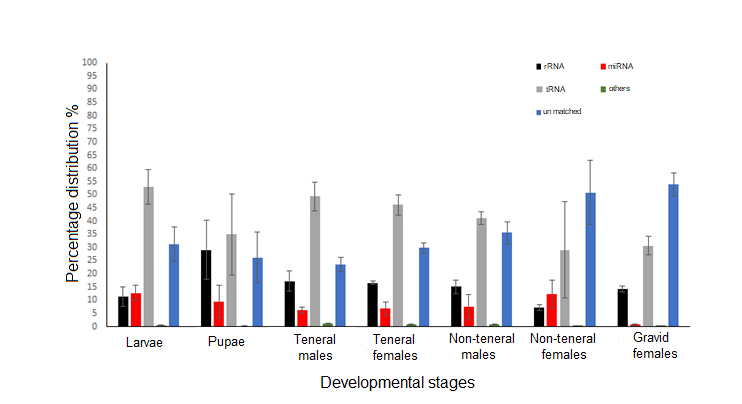


**Supplementary figure 2**: The small non-coding RNA distribution from Rfam database in the different developmental stages. Others and unmatched represent other types of small non-coding RNA establish in Rfam and unaligned reads from the libraries, respectively.
